# Social Capital and Psychological Well-Being of Chinese Immigrants in Japan

**DOI:** 10.3390/ijerph18020547

**Published:** 2021-01-11

**Authors:** Shun Gong, Peng Xu, Senhu Wang

**Affiliations:** 1 Institute of Sociology, Chinese Academy of Social Sciences, Beijing 100732, China; gongshun@cass.org.cn; 2 Department of Sociology, Zhongnan University of Economics and Law, Wuhan 430073, China; 3 Department of Sociology, National University of Singapore, Singapore 117570, Singapore

**Keywords:** bonding and bridging social capital, psychological well-being, Chinese immigrants, Japan

## Abstract

Social capital in immigrants has drawn considerable attention from social scientists. Previous studies have paid attention to how immigrants’ bonding social capital (defined as social networks with co-ethnic residents) and bridging social capital (defined as social networks with native residents) are associated with their economic achievement. However, little attention has been paid to immigrants’ different social capital’s effects on psychological well-being. Drawing data from Chinese immigrants in Japan, we examined how these Chinese immigrants assimilated into Japanese society and how their bonding and bridging social capital related to their psychological well-being. The results show that bonding social capital directly affected immigrants’ psychological well-being, whereas bridging social capital indirectly improved their psychological well-being by improving economic status. This study contributes to previous literature on how immigrants’ different social capital is related to their psychological well-being.

## 1. Introduction

The social capital theory is undoubtedly one of the most influential theories in social science. The conceptualization of social capital and its effects on society has attracted a considerable amount of interest from scholars [1,2,3]. Amid rising research discussions on social capital distinguish between “bonding” and “bridging” social capital [3,4]. The definition is based on the actors to whom the networks are connected. Bonding social capital refers to within-group connections while bridging social capital refers to between-group connections [5]. It is argued that bonding and bridging social capital have different effects on individuals’ psychological well-being and economic achievement [3,5,6]. 

Social capital in immigrants has also drawn considerable attention from researchers. When analyzing the relationship between social capital and immigrants’ adaptation in the mainstream society, most previous studies are more interested in the relationship between immigrants’ social capital and economic achievement [7,8,9,10]. For instance, according to the existing studies in the US, Europe, and Japan, immigrants’ bridging social capital are found to provide useful information and support for immigrants’ economic upward mobility [8,9,10,11,12,13]. Additionally, because immigrants are likely to confront linguistic and cultural barriers and obstacles, their bridging social capital with native residents are found more important than bonding social capital because native residents control more resources and economic opportunities in mainstream society than do immigrants’ co-ethnic residents [13]. 

Although a few scholars have paid attention to immigrants’ social capital and psychological well-being, the literature often focus more on whether different social capital affects immigrants’ psychological well-being or not [3,13]. A frequently heard conclusion is that while bonding social capital is important for immigrants’ psychological well-being, bridging social capital has no effect on immigrants’ psychological well-being. For instance, immigration studies in the US have revealed although bonding social capital provides less instrumental support; however, they may be more likely to promote better subjective well-being [12]. An empirical study regarding Brazilian immigrants’ social capital and psychological well-being confirmed that Brazilian immigrants in Japan benefited significantly from bonding social capital with their extended families in terms of improved mental health [13]. Vietnamese migrant workers’ bonding social capital in Taiwan is found to be the central to the social life of Vietnamese workers, which offered not only material but also psychological support [14]. However, there is so far no empirical research on Chinese immigrants in Japan. 

Moreover, there is little empirical evidence regarding the ways in which bonding and bridging social capital affects immigrants’ psychological well-being in general. It is therefore important to examine the mechanism of how immigrants’ bonding and bridging social capital affect their psychological well-being. Additionally, although the Chinese immigrants in Japan have rapidly increased from the 1980s and is the largest immigrant group now, little attention has been paid to Chinese immigrants’ social capital and psychological well-being. Chinese immigrants are characterized by high skill levels and educational attainment, which is consistent with the main rule of the new immigration law adopted in 1989 stating that Japan accepts only skilled immigrants and excludes unskilled ones from entry [13,15,16,17,18,19,20]. In other words, Chinese immigrants are not only the largest but also one of the most representative new waves of immigrant groups in Japan. Thus, the analysis of how Chinese immigrants’ social capital matters for their psychological may have important policy implications for Japan’s immigration policy. Finally, most previous studies have not systematically conceptualized immigrants’ bonding and bridging social capital for immigrants. For instance, although bonding social capital is generally defined as immigrants’ connection with co-ethnic peers, some studies used the number of people who can provide personal support or close friends as bonding social capital [21]. This measurement denied immigrants’ possibility to have a close relationship with native residents or other immigrant groups. 

To fill those research gaps, this study uses data from Chinese immigrants in Japan and examines how immigrants’ different social capital affect their psychological well-being. Overall, this article makes two important contributions. First, this article is the first study that focused on Chinese immigrants in Japan. Second, this article is also the first study that investigated the distinct ways in which social capital influences immigrants’ psychological well-being. For example, we studied whether immigrants’ bonding and bridging social capital can directly affect their subjective well-being, or indirectly affect their subjective well-being through improving their economic status. The structure is as follows. We begin by reviewing previous research on immigrants’ social capital and psychological well-being. Next, we present the analyses and discussing the results. Finally, we conclude by reflecting upon the implications of our results.

## 2. Materials and Methods 

### 2.1. Social Capital and Psychological Well-Being of Immigrants

A considerable number of studies have discussed the concept of social capital and its hypothesized effects. Previous scholars have offered different definitions of social capital [5,6,22,23]. For instance, Lin (2001) proposed that social capital represents the “resources embedded in a social structure that are accessed and/or mobilized in purposive actions” [23], and Putman defined social capital as the “features of social organization” and social networks [5]. 

Although different definitions of social capital have been proposed by scholars, most researchers agree that the core of social capital is the social network between actors [5]. According to this definition, social capital is divided into two types based on how they connect actors. The social capital between similar actors is “bonding social capital” and the social capital among heterogeneous group actors are “bridging social capital”. The psychological and objective benefits of bonding and bridging social capital has been widely explored from numerous perspectives; most scholars have noted the importance of bridging social capital for upward mobility for actors, whereas bonding social capital is considered to create strong norms and play a protective role in actors’ psychological well-being [5]. 

With regard to the literature in immigration, immigrants’ psychological well-being and social integration has received much scholarly and policy attention [4,7,8,9,10,11,14,24]. In particular, social scientists also often analyzed how immigrants’ bonding and bridging social capital affects their lives after migration. For example, according to straight-line assimilation theory, immigrants’ social capital with members of the host society are found to be a critical dimension of immigrants’ integration [25,26,27], whereas the segmented assimilation theory also emphasized immigrants’ bonding social capital with co-ethnic peers are also essential for immigrants’ adaptation [28]. However, most research on the relation between immigrants’ different social capital and adaption are explored from the economic perspective [8,26,29], little attention has been paid to immigrants’ psychological well-being. 

Most importantly, the existing research has not explored the mechanism in which immigrants’ different social capital affect their psychological well-being. According to previous research, we proposed that there are direct and indirect effects between immigrants’ bonding and bridging social capital and their psychological well-being. 

First, because immigrants have fewer resources in the destination countries and provide relatively little useful information, bonding social capital is less likely to improve immigrants’ income [7]. However, immigrants’ bonding social capital is greatly beneficial to immigrants’ psychological well-being, because bonding social capital will help immigrants to overcome the obstacles that stem from discrimination and cultural barriers [13,28]. Thus, the direct effects of immigrants’ bonding social capital on psychological well-being exist. 

Second, it might be reasonable that bridging social capital has an indirect effect on psychological well-being through their ability to improve income [30]. Specifically, similar to the “strength of weak ties” theory, immigrants’ bridging social capital with citizens of the host country provide useful information and support for immigrants’ upward mobility [8,9,10]. It is obviously the economic upward mobility that will improve their psychological well-being [27]. As a result, the indirect effects of immigrants’ bridging social capital on psychological well-being exist. 

Based on the ideas described above, we hypothesized the following:

**Hypothesis 1** **(H1).** *Bonding social capital directly improves Chinese immigrants’ psychological well-being in Japan, but it does not have an indirect effect from income to psychological well-being.*

**Hypothesis 2** **(H2).** *Bridging social capital does not directly improve Chinese immigrants’ psychological well-being, but it indirectly increases immigrants’ psychological well-being by improving their income.*

### 2.2. Chinese Immigrants in Japan

Due to Japan’s limited resources and space for farming, Japanese emigration to the US and South American communities, such as Brazil and Peru, was more common than immigration to Japan for many years. Immigration to Japan is divided into two waves: the first wave occurred from 1929 to the 1950s, and the second wave began in 1989 and continues to the present. The first wave of immigrants began to arrive during Japan’s colonial period, when immigrants from Japan’s colonies, such as Korea and China, migrated to Japan beginning in 1910 (and especially after 1929), mainly as forced labour [18,31]. 

**Figure 1:**
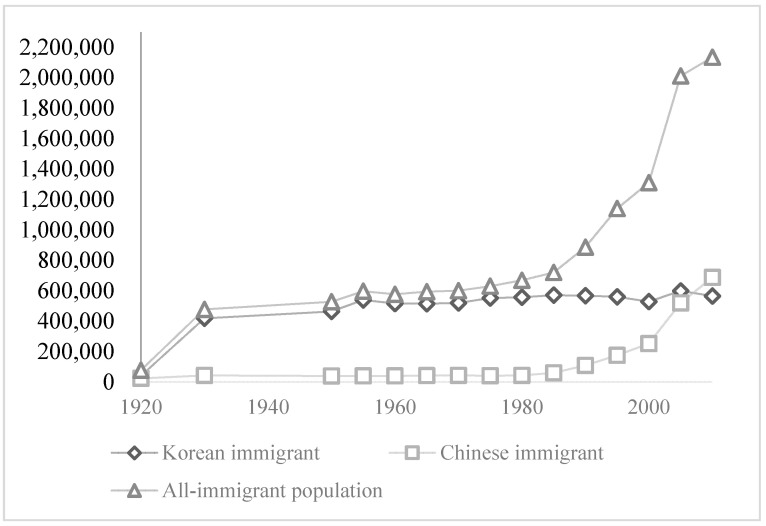
Time series trends of immigrant populations in Japan. Source: Japanese Census Data 1920–2010.

As shown in Figure 1, most of the immigrants in the first wave came from Korea. After Japan’s losses in WWII and with the passage of the 1952 Treaty of Peace with Japan in San Francisco, Japan’s new immigration law was established. This law established the framework for Japan’s post-war immigration policy, which did not encourage further settlement. The second wave of immigration did not begin until the 1980s, when, because of a labor shortage problem, Japan reopened its doors to immigrants. The second wave of immigration is characterized by its remarkable openness to skilled immigrants, whereas unskilled immigrants are still restricted from entering the country [15]. The only way unskilled immigrants can enter Japan is through the so-called “side door”. For example, Japan’s immigration law admits only less-educated “Nikkeijin”, who have Japanese ancestors, as unskilled immigrants [31]. Other unskilled immigrants who lack this special relationship with Japan can enter only through the guest-worker programme, which offers five-year visas and far lower salaries than those of guest workers’ Japanese colleagues [16].

The second wave of immigrants that began arriving the 1980s includes a high proportion of Chinese immigrants. In 2007, Chinese immigrants exceeded Korean immigrants in numbers, becoming the largest immigrant group in Japan. In addition, in contrast to unskilled Brazilian immigrants, most Chinese immigrants are the direct result of Japan’s new immigration law that was established after 1989, which shows preference for “skilled immigrants” and imposes restrictions on unskilled immigrants. Consequently, Chinese immigrants are not only the largest but also one of the most representative new waves of immigrant groups in Japan. By investigating Chinese immigrants in Japan, we can provide some answers to questions regarding how the new wave of immigrants are assimilating in Japan, which may have important implications for Japan’s immigration policy.

### 2.3. Data and Measures 

Random sampling is desirable for analysing immigrants’ social capital and psychological well-being; however, until recently, information about immigrants to Japan has not been accessible to researchers [19]. As a result, only limited randomly sampled immigrant data were available for use in our analysis. Therefore, we used a web-monitoring survey called the “Chinese Immigrant Disaster Consciousness Survey” conducted by the Leading Program at Tohoku University, Japan. The dataset was collected in February 2016, and the survey is based on responses from customers of the research company, which monitors more than 10,000 registered immigrants and 1200 of them are Chinese immigrants. Among the 1200 Chinese immigrants monitored, the survey obtained a random sample of 256 Chinese immigrants, who were sent questionnaires in Chinese. The survey received responses from a final sample of 192 Chinese immigrants, for a response rate of 74.6%. 

Because our dataset is not randomly sampled, its representativeness might be limited. However, the dataset has its own advantages for understanding Chinese immigrants’ psychological well-being. Our dataset differs from other datasets because one of its goals is to determine Chinese immigrants’ co-ethnic and native social capital, disaster awareness, and psychological well-being in Japan. Thus, this dataset offers us the ability to obtain more specific answers related to Chinese immigrants’ social capital and psychological well-being. For example, the dataset includes more than 5 dimensions of Chinese immigrants’ social capital or networks with both their Chinese peers and Japanese citizens, and the questionnaire regarding psychological well-being also includes 6 questions. 

### 2.4. Variables 

Table 1 shows the descriptive statistics of the variables involved in this study. For continuous variables, we reported the mean, standard deviation, minimum and maximum values of the variables. For categorical variables, we only reported the percentages of the different categories.

### 2.5. Dependent Variable

We utilized one of the most widely used indexes, the Kessler Psychological Distress Scale 6 (K6) [32], to measure the psychological well-being of Chinese immigrants in Japan. The K6 scale was developed with support from the U.S. government’s National Center for Health Statistics for use in the redesigned U.S. National Health Interview Survey (NHIS), which was sensitive around the threshold for the clinically significant range of the distribution of nonspecific distress. This questionnaire is organized in the following format:

The following questions ask about how you have been feeling during the past 30 days. For each question, please circle the number that best describes how often you had this feeling.

- Nervous
- Hopeless
- Restless or fidgety
- So depressed that nothing could cheer you up
- Everything was an effort
- Worthless

Answers to the questions were recorded on a 5-point scale: “1: All the time”, “2: Most of the time”, “3: Some of the time”, “4: A little of the time”, and “5: None of the time”. A higher score reflects better psychological status. Table 2 provides summary statistics of the K6 data. Because a higher score reflects better psychological status, the K6 results related to distress show that Chinese immigrants’ psychological well-being is in a better condition. Table 2 provided the summary statistics of K6 scale in our samples.

Next, using a factor analysis model, we created one variable, “immigrants’ psychological well-being”. The results of the factor analysis are shown in Table 3.

### 2.6. Independent Variables

As proposed in our hypotheses, we aim to learn about the effects of Chinese immigrants’ bonding and bridging social capital on their psychological well-being. Thus, using the social support index proposed by Wills et al. [33], we created variables describing immigrants’ bonding and bridging social capital. The questions designed by Wills et al. ask how many friends provide the following types of support:

- Help you with emotional problems
- Help you with money
- Provide you with information about jobs
- Provide companionship to you
- Understand your thoughts

The respondents were asked to self-report a specific number for how many of their Japanese and Chinese friends can provide the above forms of help. Similarly, using the factor analysis method, we created variables named “Bonding social capital” and “Bridging social capital”; the results are shown in Table 4 and Table 5, respectively.

Another independent variable was immigrants’ income. We analysed immigrants’ self-reported income using the natural logarithm of annual wages before tax deductions during the previous year.

### 2.7. Control Variables

Lastly, the present study included the following control variables: the immigrants’ age, Japanese proficiency, years since migration to Japan, gender, marital status, educational attainment, education experience in Japan, and frequency of confirming disaster information. Immigrants’ ages were calculated based on their year of birth. Their Japanese proficiency was based on a subjective evaluation of their Japanese writing, speaking, and reading abilities; here, we also performed a factor analysis to create a new variable, called “immigrants’ Japanese proficiency”, in the analysis. The results of this factor analysis are depicted in Table 6.

The number of years of residence was calculated based on the year in which the immigrants arrived in Japan. Educational attainment was a categorical variable, with 1 representing college and above and 0 referring to educational attainment at any level less than college. The ability to attain an education in the host country is important for immigrants because it not only increases the return of human capital (e.g., education) but also fosters immigrants’ assimilation [27]. We created a new variable, education experience in Japan, with 1 representing immigrants who were educated in Japan and 0 representing immigrants who were not educated in Japan. We also considered the marital status of immigrants. We used immigrants who married Japanese people as the reference group and created two other categories: “Single” and “Married but spouse is not Japanese”. Finally, the confirmation of disaster information, although it is not directly related to immigrants’ well-being in the host society, offers certain suggestions about the mental state of immigrants; those who have confirmed disaster information might occasionally be more positive or optimistic about their lives. We also controlled for this effect, with 0 representing “does not confirm”, 1 representing “more than once per month”, and 2 indicating “more than once per week”. The results of all descriptive characteristics of the variables were shown in above-mentioned Table 1.

As depicted in Table 1, compared to their bonding social capital, the Chinese immigrants’ bridging social capital was relatively low. For example, fewer Japanese than Chinese friends were reported to provide any of the five dimensions of support. However, the results also imply that Chinese immigrants, or any new wave of immigrants in Japan, do have connections with Japanese citizens, implying that they are assimilating into mainstream Japanese society and communities. 

Finally, regarding the representativeness of the dataset, more than half of the respondents were regularly employed immigrants (56.6%), as shown in Table 1, which was consistent with the overall distribution of Chinese immigrants in Japan [34]. In addition, the proportion of males was 43.8%, the average age was approximately 35 years, the average length of residence in Japan was approximately 9 years, and more than half of the respondents had been educated in Japan and had a college degree. These results were also consistent with the overall characteristics of Chinese immigrants in Japan, who tended to be relatively well educated and newcomers to Japan. The data showed no extreme biases compared to previous studies, which confirms that our data, although not derived from a nationally representative sample, can represent Chinese immigrants in Japan. 

### 2.8. Analytical Strategies and Measurements 

We evaluated how Chinese immigrants’ bonding and bridging social capital affect their psychological well-being. In the first step, we regressed Chinese immigrants’ psychological well-being on their bonding social capital and bridging social capital using an ordinary least-squares (OLS) regression model and investigated our hypotheses concerning the direct effects of bonding and bridging social capital on psychological well-being. Next, we used Structural Equation Modeling (SEM) to analyze the data, which allowed us to determine both the indirect and direct effects of immigrants’ bonding and bridging social capital on their psychological well-being.

## 3. Results

As mentioned above, using an OLS model, we first tested our hypothesis regarding how Chinese immigrants’ bonding and bridging social capital affects their psychological well-being directly. Table 7 shows the results of our OLS models with robust standard errors. Model 1 includes the independent variables for bonding social capital; Model 2 regresses immigrants’ psychological well-being on bridging social capital, and Model 3 is a full model including both bonding and bridging social capital. 

The coefficient for bonding social capital in Model 1 is positive and significant, indicating that Chinese immigrants’ bonding social capital was positively related to their psychological well-being. This result is consistent with previously reported hypotheses that bonding social capital is based on strong emotional networks and strong social norms and is thus effective at improving immigrants’ psychological well-being. Other coefficients in Model 1 show that age, Japanese proficiency, gender, residence years, education experience in Japan, confirmation of disaster information, and occupational status were not significantly related to Chinese immigrants’ psychological well-being in Japan. However, compared to those who married Japanese people, Chinese immigrants who did not marry Japanese people had relatively poor well-being. Moreover, income was positively and significantly related to Chinese immigrants’ well-being; conversely, a high education level was negatively related to immigrants’ psychological well-being.

Next, from Model 2 in Table 7, the coefficient for bridging social capital is not significantly correlated with immigrants’ psychological well-being, confirming bridging social capital does not directly increases immigrants’ psychological well-being. Furthermore, Model 3, which included the effects of both immigrants’ bonding and bridging social capital, again confirmed that only bonding social capital directly improves immigrants’ psychological well-being. 

**Figure 2:**
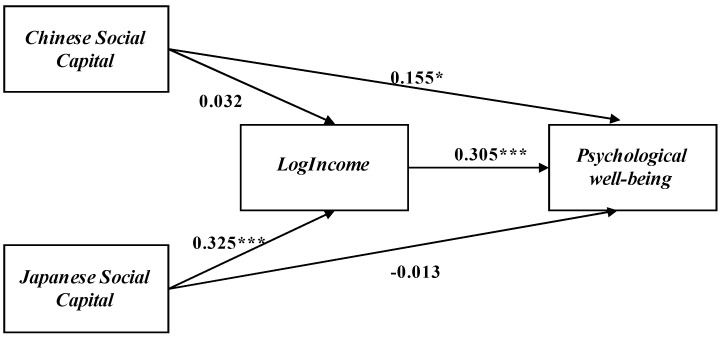
Path model for Chinese immigrants’ social capital, income, and psychological well-being. Notes: N = 188; χ^2^(df) = 117.401(25) (*p* < 0.01), CFI = 0.953, TLI = 0.901, RMSEA = 0.072, SRMR = 0.029; *** *p* < 0.001, * *p* < 0.05 (two-tailed tests); Control variables: immigrants’ income, Japanese language proficiency, gender, age, years of residence in Japan, educational attainment, education experience in Japan, marital status.

To evaluate our hypothesis regarding Chinese immigrants’ bridging social capital indirectly affect their psychological well-being, we used Structural Equation Modeling (SEM) for the analysis. The results are shown in Figure 2. 

We find that the direct and indirect relation between immigrants’ different social capital and psychological well-being exist. The bonding social capital significantly improves immigrants’ psychological well-being. However, the bridging social capital improves immigrants’ psychological indirectly through income. Specifically, we calculated the indirect effect of immigrants’ bridging social capital on their psychological well-being. The indirect effect of bridging social capital on psychological well-being was 0.325 × 0.305 = 0.099, indicating a positive relationship between immigrants’ bridging social capital and psychological well-being exist. The results show how immigrants’ different social capital affect their psychological well-being after migration. 

## 4. Conclusions

International migration has strongly manifested itself to historic highs. Many studies regarding international migration have been conducted from different perspectives [32,35,36,37,38,39,40,41,42,43,44]. Among the discussions, there is an increased interest in the issues of social capital and network resources in migration and integration research [8,9,10,11]. However, the mechanism how immigrants’ bonding and bridging social capital affect their psychological well-being is still unknown. Our paper aimed to provide a detailed mechanism regarding how immigrants’ different social capital affects their psychological well-being. 

Our analysis of a web-monitoring dataset of Chinese immigrants revealed that the bonding social capital of Chinese immigrants directly protected their psychological well-being. In contrast, although bridging social capital does not directly improve immigrants’ psychological well-being, but its indirect effect via improving income is positive and significant. Our results are in accordance with the hypothesis regarding bonding social capital’s effects on immigrants’ psychological well-being. However, we also find an indirect relation between immigrants’ bridging social capital and psychological well-being. We think this adds an important piece to understanding the role of immigrants’ different social capital in general, which helped build the detailed relationship between immigrants’ bridging social capital and psychological well-being. 

In sum, this study contributes to the literature in several ways. First, our study is distinct from most previous studies on the effects of immigrants’ social capital and psychological well-being, which mainly analyze whether different social capital affect immigrants’ psychological well-being or not. Instead, we assess the mechanism of how Chinese immigrants’ bonding and bridging social capital affects their psychological well-being. Second, the study also confirms the generalizability of the conclusions on immigrants’ social capital and psychological well-being regarding Japan’s largest and the most representative immigrant group in Japan. Finally, we have measured the bonding and bridging social capital appropriately according to theoretical definitions. 

However, the present study is not without its limitations. For example, the data used to generate our results are not a nationally representative sample of Chinese immigrants; thus, the results could suffer from some bias and may not be completely accurate. Second, there may not be a direct causal link between immigrants’ social capital and their psychological well-being and income, as immigrants with a higher income and better psychological well-being might also have higher odds of having both more co-ethnic and more native social capital. Thus, we should be cautious when concluding that immigrants’ social capital causally increases their psychological well-being. Ideally, a panel dataset will be employed in the future to investigate the causal relationship between social capital and the psychological well-being of immigrants. Finally, future research would make a significant contribution by comparing Chinese immigrants with other immigrant groups in Japan.

## Figures and Tables

**Table 1:** Description of variables.

Continuous Variables	N	Mean	S.D.	Min	Max
Psychological well-being	192	0	0.941	−3.143	1.174
Log (Income)	181	5.324	1.050	3.219	6.745
Emotional support (Japanese)	192	2.068	2.517	0	10
Help with money (Japanese)	192	1.401	1.966	0	10
Provide information about jobs (Japanese)	192	2.880	3.073	0	10
Provide companionship (Japanese)	192	2.839	3.198	0	10
Understand thoughts (Japanese)	192	2.927	3.230	0	10
Emotional support (Chinese)	192	3.995	3.137	0	10
Help with money (Chinese)	192	3.443	2.923	0	10
Provide information about jobs (Chinese)	192	4.135	3.376	0	10
Provide companionship (Chinese)	192	4.948	3.519	0	10
Understand thoughts (Chinese)	192	4.474	3.203	0	10
Age	192	34.677	8.921	21	69
Japanese proficiency	192	0	0.959	−2.731	1.073
Years since migration	192	9.401	5.581	2	28
**Categorical variables**	**Percentage (%)**				
Male	43.8				
Married to Japanese spouse	18.23				
Single	16.15				
Married but spouse is not Japanese	65.62				
Above college-level education	59.9				
Educated in Japan	56.77				
Regularly employed immigrant	56.6				
Frequency of confirming disaster information					
None	47.92				
More than once per month	36.98				
More than once per week	15.1				

**Table 2:** Summary statistics of K6 results related to feelings.

	N	Mean	S.D.	Min	Max
Nervous	192	3.938	0.925	1	5
Hopeless	192	4.219	0.894	1	5
Restless or fidgety	192	4.104	0.844	1	5
Depressed	192	4.094	0.807	1	5
Everything was an effort	192	4.125	0.859	1	5
Worthless	192	4.307	0.859	1	5

**Table 3:** Summary of results from exploratory factor analysis of psychological well-being.

Item	Factor Loading
	Identification
Nervous	0.7105
Hopeless	0.7942
Restless or fidgety	0.8185
Depressed	0.7448
Everything was an effort	0.737
Worthless	0.6992
Eigenvalue	3.392
Number of test measures	6

**Table 4:** Summary of results from exploratory factor analysis of bonding social capital.

Item	Factor Loading
	Identification
Emotional support	0.8485
Help with money	0.7886
Provide information about jobs	0.7295
Provide companionship	0.7656
Express understanding	0.815
Eigenvalue	3.125
Number of test measures	5

**Table 5:** Summary of results from exploratory factor analysis of bridging social capital.

Item	Factor Loading
	Identification
Emotional support	0.7788
Help with money	0.7737
Provide information about jobs	0.7571
Provide companionship	0.7878
Understand thoughts	0.838
Eigenvalue	3.101
Number of test measures	5

**Table 6:** Summary of results from exploratory factor analysis of Japanese proficiency.

Item	Factor Loading
	Identification
Reading	0.915
Speaking	0.93
Writing	0.866
Eigenvalue	2.452
Number of test measures	3

**Table 7:** Regression analysis of immigrants’ psychological well-being.

	Model 1	Model 2	Model 3
Age	0.010	0.010	0.010
	(0.010)	(0.011)	(0.010)
Japanese proficiency	0.075	0.110	0.083
	(0.101)	(0.101)	(0.098)
Male	−0.029	−0.021	−0.032
	(0.152)	(0.154)	(0.152)
Single	−0.784 ***	−0.730 ***	−0.820 ***
	(0.241)	(0.261)	(0.256)
Married but spouse is not Japanese	−0.274	−0.218	−0.313
	(0.173)	(0.186)	(0.197)
Years since migration	−0.015	−0.020	−0.014
	(0.017)	(0.018)	(0.018)
Above college-level education	−0.457 ***	−0.389 ***	−0.456 ***
	(0.124)	(0.127)	(0.126)
Educated in Japan	0.117	0.064	0.106
	(0.155)	(0.152)	(0.156)
Confirming disaster information: (more than once per month)	−0.094	−0.093	−0.090
	(0.139)	(0.142)	(0.140)
Confirming disaster information (more than once per week)	−0.345	−0.298	−0.325
	(0.220)	(0.228)	(0.218)
Regularly employed immigrant	0.038	0.034	0.044
	(0.144)	(0.146)	(0.145)
Log (Income)	0.282 ***	0.279 ***	0.289 ***
	(0.067)	(0.069)	(0.070)
Bonding social capital	0.183 ***		0.215 **
	(0.068)		(0.083)
Bridging social capital		0.064	−0.061
		(0.077)	(0.092)

Notes: *** *p* < 0.001, ** *p* < 0.01 (two-tailed tests).

## Data Availability

The data are not publicly available due to privacy reason.

## References

[1] Inaba A. (2007). Social Support, Care, and Social Capital. J. Welf. Sociol..

[2] Poder T.G. (2011). What is Really Social Capital? A Critical Review. Am. Sociol..

[3] Poortinga W. (2012). Community resilience and health: The role of bonding, bridging, and linking aspects of social capital. Health Place.

[4] Karki S., Mix T.L. (2018). “My Family Are Supportive…But People in My Village Mock Me”: Bonding and Bridging Capital among Women Pursuing Secondary Education in Kathmandu, Nepal. Sociol. Perspect..

[5] Putman R.D. (2001). Bowling Alone: The Collapse and Revival of American Community.

[6] Sato Y. (2003). Social Capital. Sociopedia.ISA.

[7] Kalter F., Kogan I. (2014). Migrant Networks and Labor Market Integration of Immigrants from the Former Soviet Union in Germany. Soc. Forces.

[8] Lancee B. (2010). The Economic Returns of Immigrants’ Bonding and Bridging Social Capital: The Case of the Netherlands. Int. Migr. Rev..

[9] Lancee B. (2012). The economic returns of bonding and bridging social capital for immigrant men in Germany. Ethn. Racial Stud..

[10] Lancee B. (2015). Job search methods and immigrant earnings: A longitudinal analysis of the role of bridging social capital. Ethnicities.

[11] Aguilera M.B. (2005). The Impact of Social Capital on The Earnings of Puerto Rican Migrants. Sociol. Q..

[12] Portes A., Rumbaut R.G. (2006). Immigrant America: A Portrait.

[13] Takenoshita H. (2015). Social Capital and Mental Health among Brazilian Immigrants in Japan. Int. J. Jpn. Sociol..

[14] Hoang L.A. (2015). Vietnamese migrant networks in Taiwan: The curse and boon of social capital. Ethn. Racial Stud..

[15] Cornelius W.A., Tsuda T., Cornelius A.W., Tsuda T., Martin L.P., Hollifield F.J. (2004). Japan: Government policy, immigrant reality. Controlling Immigration: A Global Perspective.

[16] Gong S. (2017). Are the Consequences of Experiencing Discrimination the same for Immigrants of Differing Socio-Economic Status in Japan?. Int. Migr..

[17] Green D. As Its Population Ages, Japan Quietly Turns to Immigration. The Online Journal of Migration Policy Institute. https://www.migrationpolicy.org/article/its-population-ages-japan-quietly-turns-immigration.

[18] Holbrow H.J., Nagayoshi K. (2018). Economic Integration of Skilled Migrants in Japan: The Role of Employment Practices. Int. Migr. Rev..

[19] Takenaka A., Nakamuro M., Ishida K. (2016). Negative Assimilation: How Immigrants Experience Economic Mobility in Japan. Int. Migr. Rev..

[20] Takenoshita H. (2013). Labour market flexibilisation and the disadvantages of immigrant employment: Japanese-Brazilian immi-grants in Japan. J. Ethn. Migr. Stud..

[21] Tegegne M.A., Glanville J.L. (2018). The Immigrant-Native Gap in Subjective Well-Being in Western European Countries: Assessing the Role of Social Capital. Int. Migr. Rev..

[22] Coleman J.S. (2000). Social Capital in the Creation of Human Capital. Knowl. Soc. Cap..

[23] Lin N. (2001). Social Capital: A Theory of Social Structure and Action.

[24] Kessler R.C., Barker P.R., Colpe L.J., Epstein J.F., Gfroerer J.C., Hiripi E., Howes M.J., Normand S.-L.T., Manderscheid R.W., Walters E.E. (2003). Screening for Serious Mental Illness in the General Population. Arch. Gen. Psychiatry.

[25] Alba R., Nee V. (2003). Remaking the American Mainstream Assimilation and Contemporary Immigration.

[26] Waters M.C., Jiménez T.R. (2005). Assessing Immigrant Assimilation: New Empirical and Theoretical Challenges. Annu. Rev. Sociol..

[27] Zeng Z., Xie Y. (2004). Asian-Americans’ earnings disadvantage reexamined: The role of place of education. Am. J. Sociol..

[28] Portes A., Fernández-Kelly P., Haller W. (2009). The Adaptation of the Immigrant Second Generation in America: A Theoretical Overview and Recent Evidence. J. Ethn. Migr. Stud..

[29] Villalonga-Olives E., Kawachi I. (2017). The dark side of social capital: A systematic review of the negative health effects of social capital. Soc. Sci. Med..

[30] Burt R.S., Lin N., Cook K., Burt R.S. (2001). Structural holes versus network structure as social capital. Social Capital: Theory and Research.

[31] Lie J. (2001). Multiethnic Japan.

[32] Wang S., Coulter R. (2019). Exploring Ethnic and Generational Differences in Gender Role Attitudes among Immigrant Populations in Britain: The Role of Neighborhood Ethnic Composition. Int. Migr. Rev..

[33] Wills T.A., Cleary S.D. (1996). How are social support effects mediated: A test for parental support and adolescent substance use. J. Personal. Soc. Psychol..

[34] Japan Statistic Burea (2015). Japan Statistical Yearbook.

[35] Gong S., Nagayoshi K. (2019). Japanese Attitudes Toward China and the United States: A Sociological Analysis. Chin. Sociol. Rev..

[36] Wang S., Mak H.-W. (2020). Generational health improvement or decline? Exploring generational differences of British ethnic mi-norities in six physical health outcomes. Ethn. Health.

[37] Liang Y., Wei Y., Wang S., Gong S., Dong Y., Gao L., Wang S., Zhou Y., Ye N. (2019). Impact of International Immigration on Life Satisfaction of Local Residents in England: Exploring the Differentiated Relationships across Socioeconomic Gradients. Am. J. Health Behav..

[38] Wang S., Li S. (2019). Exploring Generational Differences of British Ethnic Minorities in Smoking Behavior, Frequency of Alcohol Consumption, and Dietary Style. Int. J. Environ. Res. Public Health.

[39] Wang S. (2019). The Role of Gender Role Attitudes and Immigrant Generation in Ethnic Minority Women’s Labor Force Participation in Britain. Sex Roles.

[40] Wang S., Hu Y. (2019). Migration and health in China: Linking sending and host societies. Popul. Space Place.

[41] Yan Y., Wang S., Zhou W., Wang S., Gong S. (2019). Does Immigrant Generation Matter? Re-examining the Ethnic Density Effects on Mental Health of Ethnic Minorities in Britain. Am. J. Health Behav..

[42] Wang S. (2019). Cultural and Social Integration of British Immigrants and Ethnic Minorities: Exploring Ethnic and Generational Dif-ferences in Gender Role Attitudes, Social Networks, Neighborhood Attachment and Work Values. Doctoral Thesis.

[43] Wang S. (2018). Inching Up and Socio-economic Differentiation: Exploring Self-rated Health of China’s Rural-to-Urban Migrants from 2005 to 2015. Am. J. Health Behav..

[44] Wang S., Mak H.W., Fancourt D. (2020). Arts, mental distress, mental health functioning & life satisfaction: Fixed-effects analyses of a nationally-representative panel study. BMC Public Health.

